# Impact of Type 1 Diabetes on Testicular Microtubule Dynamics, Sperm Physiology, and Male Reproductive Health in Rat †

**DOI:** 10.3390/ijms26104579

**Published:** 2025-05-10

**Authors:** Alessandra Biasi, Maria Rosaria Ambruosi, Maria Zelinda Romano, Serena Boccella, Sara Falvo, Francesca Guida, Francesco Aniello, Sabatino Maione, Massimo Venditti, Sergio Minucci

**Affiliations:** 1 Dipartimento di Medicina Sperimentale, Università degli Studi della Campania “Luigi Vanvitelli”, 80138 Napoli, Italy; alessandra.biasi@unicampania.it (A.B.); mariarosaria.ambruosi@unicampania.it (M.R.A.); mariazelinda.romano@unicampania.it (M.Z.R.); boccellaserena@gmail.com (S.B.); francesca.guida@unicampania.it (F.G.); sabatino.maione@unicampania.it (S.M.); sergio.minucci@unicampania.it (S.M.); 2 Dipartimento di Science e Tecnologie Ambientali, Biologiche e Farmaceutiche, Università degli Studi della Campania “Luigi Vanvitelli”, 81100 Caserta, Italy; sara.falvo@unicampania.it; 3 Dipartimento di Biologia, Università degli Studi di Napoli “Federico II”, 80126 Napoli, Italy; faniello@unina.it

**Keywords:** oxidative stress, spermatogenesis, microtubules, sperm quality, sperm motility, PREP, RSPH6A, type 1 diabetes

## Abstract

Type 1 diabetes (T1D) is a chronic metabolic disease defined by sustained hyperglycemia, leading to oxidative stress (OS) and systemic complications, including male subfertility. This study investigates the potential impact of T1D-induced OS on microtubule (MTs) dynamics and microtubule-associated proteins (MAPs) in the testis and spermatozoa (SPZ). Using a streptozotocin-induced T1D rat model, we examined the expression and localization of key MAPs, including Microtubule Affinity-Regulating Kinase 4 (MARK4), Microtubule-Associated Protein 1A (MAP1A), Dynein Light Chain LC8-Type 1 (DYNLL1), Prolyl Endopeptidase (PREP), and Radial Spoke Head 6 Homolog A (RSPH6A), alongside sperm functional parameters. Our findings showed that T1D significantly impaired the expression and distribution of these proteins, which may affect MTs organization and be associated with cytoskeletal disorganization, and impaired germ cell differentiation. Moreover, T1D rats exhibited reduced sperm count, viability, and motility, accompanied by increased DNA fragmentation and chromatin defects. Elevated levels of 4-hydroxy-2-nonenal (4-HNE), a marker of OS, were detected in SPZ, particularly in the acrosome and flagellum, correlating with mitochondrial dysfunction and ATP depletion. Additionally, decreased intracellular Ca^2+^ levels, downregulation of Cation Channel of Sperm (CATSPER) and Voltage-Dependent Anion Channel 3 (VDAC3), and altered tubulin acetylation, possibly due to imbalanced Alpha-Tubulin N-Acetyltransferase 1 (ATAT1) and Histone Deacetylase 6 (HDAC6) expression, were also associated with impaired sperm motility. The combined data suggest that T1D-induced OS is linked to disrupted MTs dynamics, which may contribute to testicular dysfunction and reduced sperm quality, potentially affecting male fertility. A better understanding of these associations may support the development of therapeutic strategies to mitigate the reproductive consequences of T1D and improve male fertility outcomes.

## 1. Introduction

For successful reproduction, the production and differentiation of good-quality gametes are of primary importance. In males, gametogenesis occurs in the seminiferous epithelium (SE) of the testis, which oversees the production of about 10–70 million spermatozoa (SPZ) per day in rodents, and about 200 million in men [1]. For this, massive but tightly coordinated cellular activities occur in the testicular SE to gather such considerable production. These events include the (I) maintenance and proliferation of spermatogonial stem cells and undifferentiated spermatogonia (SPG) through mitosis, (II) production of haploid spermatids (SPT) via meiosis I/II, (III) post-meiotic differentiation of round/elongated SPT into mature SPZ via spermiogenesis, (IV) release of SPZ into the lumen of the tubules [2]. Apart from germ cells (GCs) in various stages of differentiation, in the SE reside the Sertoli cells (SCs), which form a polarized cell layer that supports spermatogenesis. Each step of such an intricate developmental program is finely regulated by the activation or repression of numerous genes, in many cases testis-specific, in each cell type and stage [3,4,5,6,7,8,9]. Several events occurring during male gametogenesis depend on continuous remodeling of the cytoskeletal architecture, particularly in the microtubules (MTs), of all the involved cells. Worth remembering, MTs are hollow tubes of ∼25 nm in diameter made up of α- and β-tubulin (TUB) heterodimers involved in key cellular processes, including cell morphology, mitosis, intracellular trafficking, and movement [10,11]. Besides forming the spindle for mitosis and meiosis, MTs also regulate fundamental aspects, including spermiogenesis and SPT transport towards the lumen, as well as SPZ physiology. Another fundamental aspect of spermiogenesis is the formation of the flagella, a structure possessing a typical 9 + 2 central MTs core, the axoneme that, working in concert with many other proteins (dynein, radial spokes, and so on), is the basis of proper sperm motility and, therefore, fertilization capability [12,13,14,15]. Finally, SPT translocation in the SE occurs via MTs-based transport of the apical ectoplasmic specialization (ES), a testis-specific actin-based junction type at the interface between SCs and elongating/elongated SPT. It has been proposed that the role of MTs, which run parallel to the longitudinal SC axis, in SPT movement is to form rail-track structures, gliding the whole ES together with the attached SPT, supported by motor proteins [16,17,18].

In this complex scenario, it is evident that the highly specialized function and dynamics of MTs depend on a plethora of microtubule-associated proteins (MAPs), comprising molecular motors, cross-linking MAPs, severing factors, nucleators, and modulators of MTs dynamics [19,20,21,22,23,24]. The importance of the role of MAPs also relies on the fact that their deletion, inactivation, mutation, and/or genetic variation of regulatory genes may lead to infertility and/or subfertility in humans and/or rodents [25]; however, the exact molecular mechanisms are still to be completely elucidated. In recent years, the study of MTs and cytoskeletal dynamics in spermatogenesis has been developed based on the use of toxicant models, such as the endocrine disrupter and oxidative stress (OS) inducers cadmium, PFOS, and the male contraceptive adjudin [26,27,28].

Among the systemic diseases that impair male fertility, type 1 diabetes (T1D) has emerged as a significant contributor to testicular dysfunction and subfertility. Numerous studies have demonstrated that T1D adversely affects the hypothalamic–pituitary–gonadal axis and causes both structural and functional alterations in the testes [29,30,31]. Chronic hyperglycemia is associated with increased OS, inflammation, and apoptosis in the testicular microenvironment, leading to disrupted spermatogenesis and poor sperm quality [32,33]. In experimental models, T1D has been shown to reduce the number of SPG, spermatocytes (SPC), and SPT in the SE, along with decreased seminiferous tubule diameter and degeneration of GCs [34]. Moreover, diabetic rats exhibit significantly lower sperm counts, reduced motility, and abnormal morphology, which correlate with increased levels of lipid peroxidation and decreased antioxidant enzyme activity in the testes [35]. Consistent with these findings, in our recent study, using a rat model of T1D, we confirmed the negative impact of diabetes on testicular function [36]. Specifically, we demonstrated that T1D rats showed altered blood–testis barrier (BTB) integrity, as highlighted by the dysregulated expression and localization of several markers, including Van Gogh-like protein 2 (VANGL2), a planar cell polarity protein that modulates the distribution of actin and MTs elements in SCs cytoplasm [36,37,38].

Given the susceptibility of MTs to oxidative and metabolic stress, it is plausible that diabetes-induced cytoskeletal disorganization may involve alterations in MAPs expression. Although omics-based investigations of diabetic complications are expanding, datasets specifically addressing MAPs in the testis or SPZ remain limited [39,40,41,42]. This study aims to bridge this gap by characterizing molecular and functional alterations in a T1D rat model, integrating cytoskeletal markers with OS-related outcomes. To this purpose, we focused our attention on testicular MTs dynamics and the involvement of several MAPs, namely Microtubule Affinity-Regulating Kinase 4 (MARK4) [43,44], Microtubule-Associated Protein 1A (MAP1A) [45], Dynein Light Chain LC8-Type 1 (DYNLL1) [46], Prolyl Endopeptidase (PREP) [47,48], and Radial Spoke Head 6 Homolog A (RSPH6A) [49,50,51]. Finally, the analysis was also extended to mature SPZ, by evaluating several canonical sperm parameters, including mitochondrial function, calcium (Ca^2+^) levels, and proteins regulating sperm motility and physiology.

## 2. Results

### 2.1. Effect of T1D on MARK4, MAP1A, and DYNLL1

**Figure 1:**
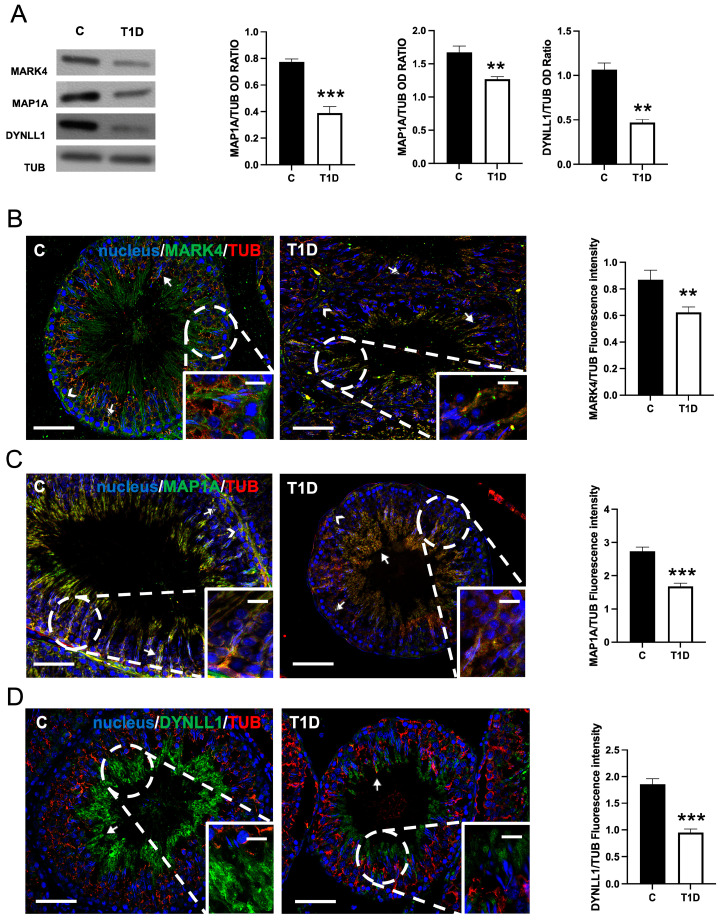
The analysis of Microtubule Affinity-Regulating Kinase 4 (MARK4), Microtubule-Associated Protein 1A (MAP1A), Dynein Light Chain LC8-Type 1 (DYNLL1) protein levels and localization in control and type 1 diabetes (T1D) rat testis. (**A**) Western blot (WB) analysis of MARK4, MAP1A, DYNLL1, and α-TUBULIN (TUB) protein levels in control and T1D rat testis. The histograms show their relative protein level. Data were normalized with TUB and reported as optical density (OD) ratio. (**B**–**D**) The immunofluorescence (IF) analysis of MARK4 (green, B), MAP1A (green, C), DYNLL1 (green, D), and TUB (red) in the testes of T1D and control animals. Slides were counterstained with 4′,6-diamidino-2-phenylindole (DAPI) fluorescent nuclear staining (blue). The scale bars represent 20 μm and 10 μm in the insets. Striped arrows: spermatocytes (SPC); arrows: spermatids (SPT); and arrowheads: Sertoli cells (SCs). Histograms show the quantification of MARK4, MAP1A1, and DYNLL1 fluorescence signal intensity; data were normalized with TUB signal. All the values are expressed as means ± standard error of the mean (SEM) from five animals in each group. **: *p* < 0.01; ***: *p* < 0.001. Each experiment was performed in triplicate.

Figure 1 shows the effects of T1D on testicular MARK4, MAP1A, and DYNLL1 expression and localization. Western blot (WB) analysis revealed that MARK4 (*p* < 0.001), MAP1A (*p* < 0.01), and DYNLL1 (*p* < 0.01) protein levels were significantly lower in the T1D group, as compared to the controls (Figure 1A).

Immunofluorescence (IF) analysis showed that MARK4 (Figure 1B) was detected, in the control testis, in SPC (striped arrows) and, predominantly, within the SPT heads (arrows) and the cytoplasmic extensions of SCs (arrowheads; inset), where a clear co-localization with TUB was highlighted by the yellow-orange intermediate tint. In testis sections from T1D rats, the localization pattern of MARK4 was like that of the control, but a clear decrease in staining was evident.

MAP1A (Figure 1C) localized, in the control testis, in SPC (striped arrows), in elongated SPT (arrows), whose tail extends towards the lumen showing a clear striped conformation, as well as in the SCs cytoplasmic protrusions in co-localization with TUB (arrowheads; inset), as highlighted by the overlapping yellow-orange signal. In the T1D group, a weaker signal (*p* < 0.001) and a general “disorganization” of MAP1A distribution in the SPT cytoplasm (arrows) and SCs protrusions (arrowheads; inset) were observed.

Finally, as for DYNLL1 (Figure 1D), it localized in the cytoplasm of elongating SPT (arrows; insets) in both groups; however, a weaker fluorescent signal in T1D as compared to the controls was observed (*p* < 0.001).

### 2.2. Effect of T1D on PREP and RSPH6A

**Figure 2:**
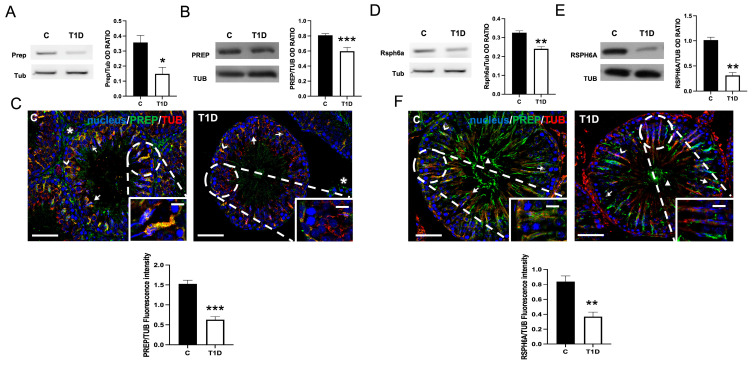
The analysis of Prolyl Endopeptidase (PREP) and Radial Spoke Head 6 Homolog A (RSPH6A) expression and localization in control and T1D rat testis. (**A**,**D**) Agarose gel electrophoresis of RT-PCR products showing the expression of *Prep* and *Rsph6a*, respectively, in testes of controls and T1D animals. The histograms show their relative mRNA levels. Data were normalized with *Tub* and reported as OD ratio. (**B**,**E**) WB analysis of PREP, RSPH6A, and TUB protein levels in control and T1D rat testis. Histograms show their relative protein level. Data were normalized with TUB and reported as OD ratio. (**C**,**F**) IF analysis of PREP (green), RSPH6A (green), and TUB (red) in the testes of T1D and control animals. The slides were counterstained with DAPI-fluorescent nuclear staining (blue). The scale bars represent 20 μm and 10 μm in the insets. Striped arrows: SPC; arrows: SPT; arrowheads: SCs; triangle: luminal spermatozoa (SPZ); and asterisks: Leydig cells. The histograms show the quantification of PREP and RSPH6A fluorescence signal intensity; data were normalized with TUB signal. All the values are expressed as means ± SEM from five animals in each group. *: *p* < 0.05; **: *p* < 0.01; ***: *p* < 0.001. Each experiment was performed in triplicate.

Figure 2 shows the effects of T1D on testicular PREP and RSPH6A expression and localization. The mRNA (*p* < 0.05; Figure 2A) and protein (*p* < 0.001; Figure 2B) levels of PREP were downregulated in the T1D group, as compared to the controls.

PREP immunolocalization (Figure 2C) showed that in the control, it localized in SPC (striped arrows) and, principally, within the SPT (arrows) and the cytoplasmic extensions of SCs (arrowheads; inset), where a clear co-localization with TUB was highlighted by the merged yellow-orange fluorescence; finally, the signal also appeared into the interstitial Leydig cells (asterisks). In testis sections from diabetic animals, the localization pattern of PREP was comparable to that of the control, but a marked reduction in staining intensity was observed (*p* < 0.001), particularly in SPT (arrowheads) and the SC cytoplasm (striped arrows; inset).

As for RSPH6A, its mRNA (*p* < 0.01; Figure 2D) and protein (*p* < 0.01; Figure 2E) levels were significantly lower in T1D animals compared to controls. IF analysis on RSPH6A (Figure 2F) showed its localization, in the control testis, in SPT (arrows), and the SC cytoplasmic protrusions in co-localization with TUB (arrowheads; inset), as shown by the yellow-orange intermediate color; finally, the signal was also present in the tail of luminal SPZ (triangle). In testis sections from diabetic animals, the RSPH6A localization pattern was like that of the control, but a noticeable decrease in staining intensity was evident (*p* < 0.01), particularly in the luminal SPZ (triangle).

### 2.3. Effect of T1D on Sperm Parameters and Quality

The effects of T1D were extended on gamete physiology. Firstly, Table 1 shows the results of the analysis of the main sperm parameters. Data revealed significant differences between T1D and control groups in sperm number (*p* < 0.05), viability (*p* < 0.01), morphology (*p* < 0.001), and motility (*p* < 0.001).

**Figure 3:**
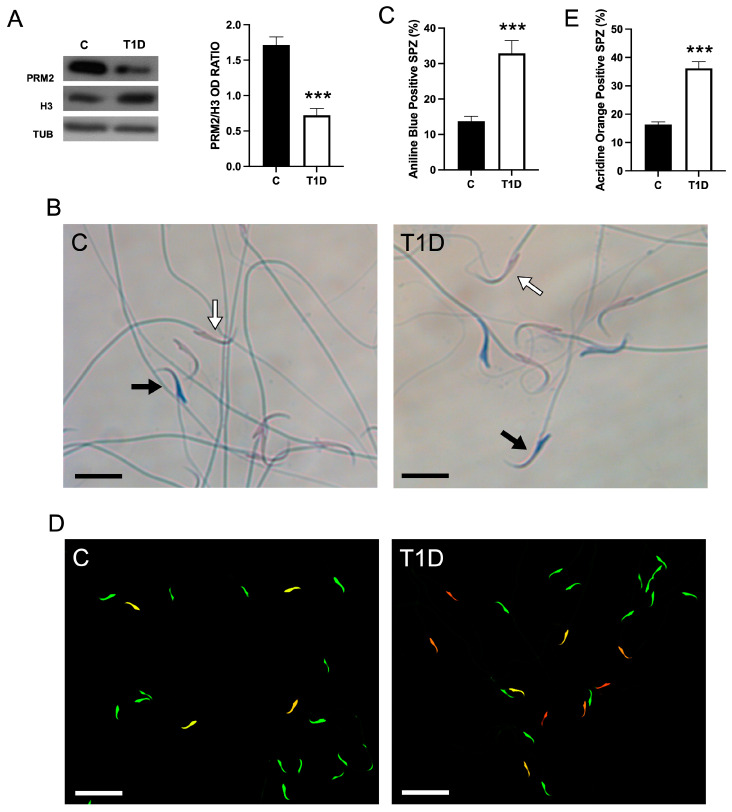
SPZ quality in control and T1D rat testis. (**A**) WB analysis of Protamine 2 (PRM2), Histone 3 (H3), and TUB in SPZ of controls and T1D animals. The histogram shows the PRM2/H3 ratio; data were normalized with TUB and reported as OD ratio. (**B**,**C**) Aniline blue (AB) staining highlights histone (white arrows) and protamine (black arrow) content in the SPZ of control and T1D animals. The histogram shows the % of AB-positive cells. (**D**,**E**) Acridine orange (AO) staining highlights the SPZ with damaged DNA (yellow/orange/red) with respect to those with intact DNA (green). The histogram shows the percentage of SPZ with damaged DNA. The scale bars represent 20 μm. ***: *p* < 0.001. Each experiment was performed in triplicate.

To assess the chromatin defects, and DNA integrity in sperm, aniline blue (AB) and acridine orange (AO) staining were performed (Figure 3).

The results showed an increased percentage of SPZ showing a dark blue head (indicating a higher content of lysine-rich histones) as compared to the controls (*p* < 0.001; Figure 3B,C). These data were supported by WB analysis of Histone 3 (H3) and Protamine 2 (PRM2), showing histone and protamine content markers, respectively. The results indicated a lower PRM2/H3 ratio in T1D rats as compared to the controls (*p* < 0.001; Figure 3A), suggesting the presence of immature SPZ characterized by mistakes in histone–protamine exchange [52].

Assessment via AO staining demonstrated a significant elevation in SPZ displaying yellow to red fluorescence in the head, suggesting DNA damage in T1D samples as compared to the control (*p* < 0.01; Figure 3D,E).

### 2.4. Effect of T1D on Sperm OS, Mitochondrial Mass, and Apoptotic Rate

**Figure 4:**
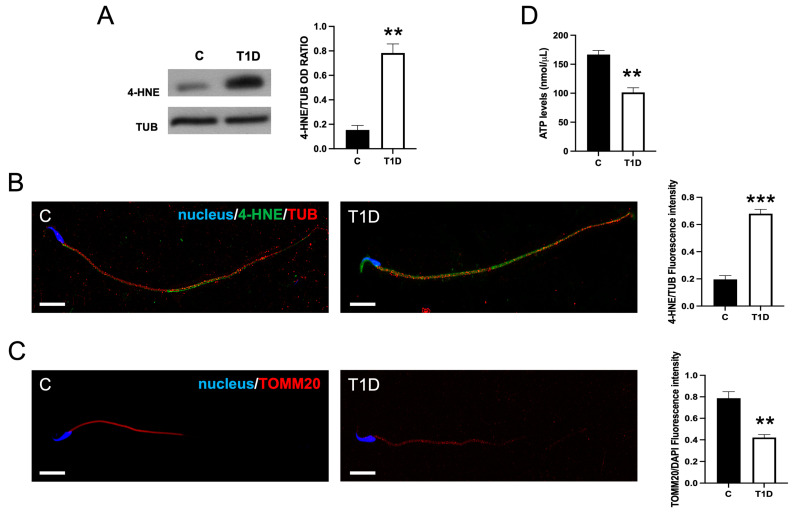
The oxidative stress and mitochondria function in SPZ of control and T1D animals. (**A**) WB analysis of 4-hydroxy-2-nonenal (4-HNE) protein levels in SPZ of control and T1D animals. The histogram shows its relative levels; data were normalized with TUB and reported as OD ratio. (**B**) 4-HNE (green) and TUB (red) immunolocalization. The slides were counterstained with DAPI-fluorescent nuclear staining (blue). The images were captured at ×40 (scale bars = 10 µm). The histogram shows the quantification of 4-HNE fluorescence signal intensity. (**C**) The ATP content in the SPZ of controls and T1D animals. (**D**) Translocase of Outer Mitochondrial Membrane 20 (TOMM20; red) immunolocalization in SPZ. The images were captured at ×40 magnification (scale bars = 10 µm). The histogram shows the quantification of TOMM20 fluorescence signal intensity. All values are expressed as means ± SEM from five animals in each group. **: *p* < 0.01; ***: *p* < 0.001. Each experiment was performed in triplicate.

To evaluate cellular OS, which is one of the major mechanisms associated with the pathophysiology of T1D [53], we evaluated the levels and localization in sperm of 4-hydroxy-2-nonenal (4-HNE), a marker of oxidative damage which forms protein adducts through covalent binding. In T1D rats, WB analysis showed markedly higher 4-HNE levels in sperm than in controls (*p* < 0.01; Figure 4A).

The IF analysis showed that the 4-HNE signal was barely visible in the tail of control SPZ, whereas, in the T1D group, it appeared stronger not only at the flagellar level but also in the head, in the proximity of the acrosomal region. An increased 4-HNE fluorescence intensity was detected in T1D samples when compared to the control group (*p* < 0.01; Figure 4B).

As one of the main sources of reactive oxygen species (ROS) hyperproduction is mitochondria, we investigated mitochondrial mass and ATP production, both of which serve as key indicators of mitochondrial integrity. The results indicated that T1D decreased the fluorescence intensity of Translocase of Outer Mitochondrial Membrane 20 (TOMM20; *p* < 0.01), a component of the TOM complex, suggesting possible mitochondrial alterations (Figure 4C). A concurrent decrease in ATP synthesis was also observed in the SPZ of diabetic rats as compared to the controls (*p* < 0.01; Figure 4D).

**Figure 5:**
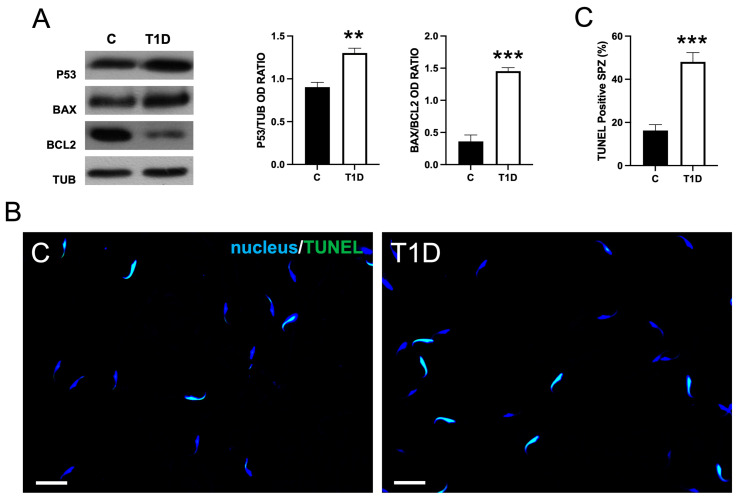
The analysis of apoptotic rate in control and T1D SPZ. (**A**) The WB analysis of P53, BAX, and BCL-2 in SPZ from control and T1D rats. The histograms show the P53 relative protein levels and BAX/BCL-2 ratio, data were normalized with TUB and reported as OD ratio (**B**) The determination of apoptotic cells through the detection of TUNEL-positive SPZ (green). The slides were counterstained with DAPI-fluorescent nuclear staining (blue). The images were captured at ×20 (scale bars = 20 µm) magnification. (**C**) A histogram showing the percentage of TUNEL-positive SPZ. All the values are expressed as means ± SEM from five animals in each group. **: *p* < 0.01; ***: *p* < 0.001.

As shown in Figure 5, T1D significantly affected the apoptotic rate of SPZ. WB data revealed an increased expression in P53 levels (*p* < 0.01), and BAX/BCL-2 ratio (*p* < 0.001) in the T1D group as compared to the control (Figure 5A). In support of these data, a Terminal deoxynucleotidyl transferase dUTP Nick End Labeling (TUNEL) assay was performed (Figure 5B); data showed an increased percentage of TUNEL-positive SPZ (*p* < 0.001; Figure 5C) in diabetic rats as compared to the controls.

### 2.5. Effect of T1D on Sperm Ca^2+^ Level

**Figure 6:**
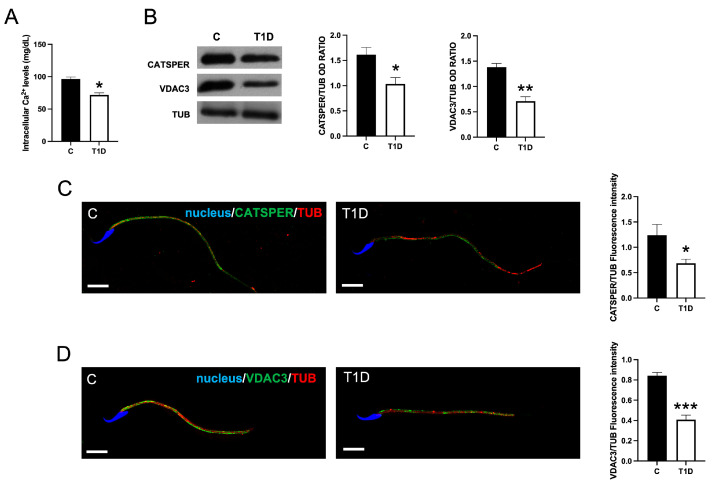
The analysis of Ca^2+^ dysregulation in control and T1D SPZ. (**A**) The intracellular Ca^2+^ levels in SPZ from control and T1D rats. (**B**). The WB analysis of Cation Channel of Sperm (CATSPER) and Voltage-Dependent Anion Channel 3 (VDAC3) in SPZ from control and T1D rats. The histograms show the relative protein levels; data were normalized with TUB and reported as OD ratio. (**C**,**D**) CATSPER (green, C), VDAC3 (green, D), and TUB (red) immunolocalization. The slides were counterstained with DAPI-fluorescent nuclear staining (blue). The images were captured at ×40 (scale bars = 10 µm) magnification. The histograms show the quantification of the fluorescence signal intensity. All values are expressed as means ± SEM from five animals in each group. *: *p* < 0.05. **: *p* < 0.01; ***: *p* < 0.001. Each experiment was performed in triplicate.

T1D reduced the intracellular Ca^2+^ levels in T1D sperm, as compared to the controls (*p* < 0.05; Figure 6A).

As confirmation of these data, WB and IF analyses on Cation Channel of Sperm (CATSPER) and Voltage-Dependent Anion Channel 3 (VDAC3)—the two main cationic channels involved in Ca^2+^ entry in the cells—were performed. WB analysis (Figure 6B) showed that both CATSPER (*p* < 0.05) and VDAC3 (*p* < 0.01) protein levels decreased in T1D as compared to the controls. These observations were consistent with the results of the IF analysis. In control sperm, CATSPER (Figure 6C) and VDAC3 (Figure 6D) localized in the flagellum of sperm, in clear co-localization with TUB, as highlighted by the overlapping yellow-orange signal. However, in the T1D group, both CATSPER (*p* < 0.05) and VDAC3 (*p* < 0.001) fluorescent intensity appeared reduced as compared to the controls.

### 2.6. Effect of T1D on Sperm Motility-Associated Factors

As the evaluation of sperm parameters revealed a decreased sperm motility in T1D rats, we decided to further explore this aspect by the analysis of acetylated tubulin (K-TUB), a well-known marker of sperm motility [54], along with Alpha-Tubulin N-Acetyltransferase 1 (ATAT1) and Histone Deacetylase 6 (HDAC), the main TUB acetyltransferase [55] and deacetylase [56], respectively.

**Figure 7:**
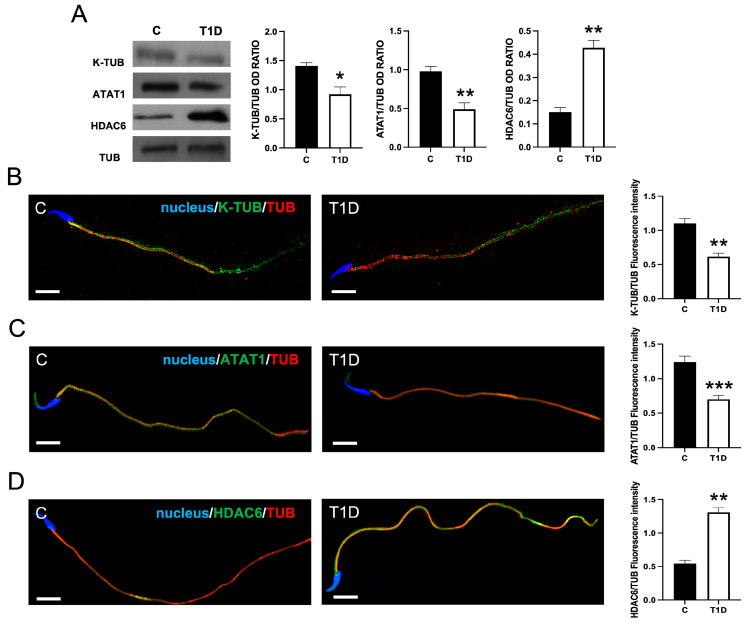
The analysis of acetylated tubulin (K-TUB), Alpha-Tubulin N-Acetyltransferase 1 (ATAT1), and Histone Deacetylase 6 (HDAC6) in control and T1D SPZ. (**A**) The WB analysis of K-TUB, ATAT1, and HDAC6 in SPZ from control and T1D rats. The histograms show their relative protein levels; data were normalized with TUB and reported as OD ratio. (**B**–**D**) K-TUB (green, B), ATAT1 (green, C), HDAC6 (green, D), and TUB (red) immunolocalization. The slides were counterstained with DAPI-fluorescent nuclear staining (blue). The images were captured at ×40 (scale bars = 10 µm). The histograms show the quantification of the fluorescence signal intensity. All values are expressed as means ± SEM from five animals in each group. *: *p* < 0.05. **: *p* < 0.01; ***: *p* < 0.001. Each experiment was performed in triplicate.

The WB analysis showed that K-TUB and ATAT1 protein levels in T1D animals were lower as compared to the controls (*p* < 0.05, and *p* < 0.01, respectively; Figure 7A); on the contrary, the HDAC6 protein level appeared to be higher in T1D sperm as compared to the controls (*p* < 0.01).

The above data were confirmed by an IF analysis performed on the three proteins. K-TUB (Figure 7B), ATAT1 (Figure 7C), and HDAC6 (Figure 7D) were exclusively expressed in the tail of sperm, in clear co-localization with TUB, as indicated by the intermediate yellow-orange signal resulting from red/green overlay. However, in T1D sperm, both K-TUB (*p* < 0.01) and ATAT1 (*p* < 0.001) fluorescent intensity was lower as compared to the controls, whereas HDAC6 signal intensity was higher in T1D (*p* < 0.001) as compared to the controls.

### 2.7. Effect of T1D on MAPs in SPZ

**Figure 8:**
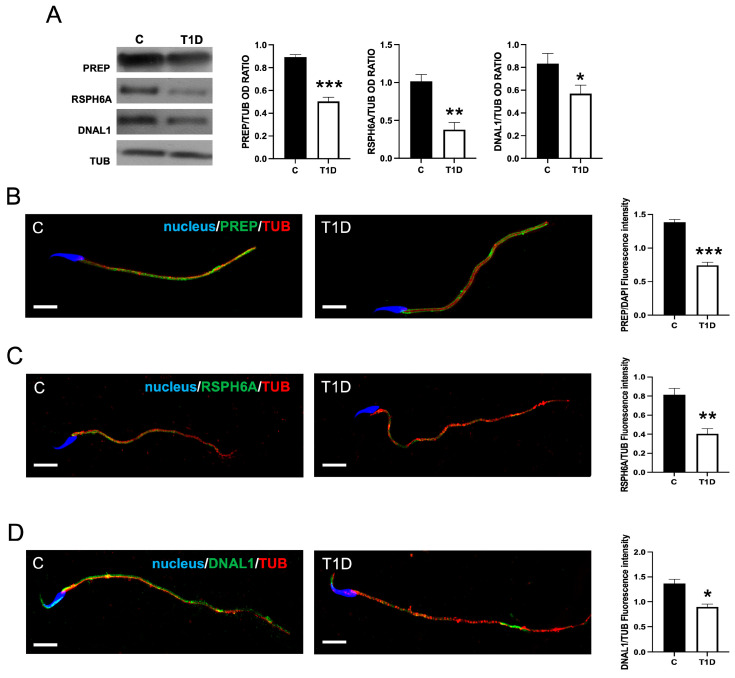
The analysis of PREP, RSPH6A, and Dynein Axonemal Light Chain 1 (DNAL1) in control and T1D SPZ. (**A**) The WB analysis of PREP, RSPH6A, and DNAL1 in SPZ from control and T1D rats. The histograms show their relative protein levels; data were normalized with TUB and reported as OD ratio. (**B**–**D**) PREP (green, B), RSPH6A (green, C), DNAL1 (green, D), and TUB (red) immunolocalization. The slides were counterstained with DAPI-fluorescent nuclear staining (blue). The images were captured at ×40 (scale bars = 10 µm). The histograms show the quantification of the fluorescence signal intensity. All values are expressed as means ± SEM from five animals in each group. *: *p* < 0.05. **: *p* < 0.01; ***: *p* < 0.001. Each experiment was performed in triplicate.

Finally, the analysis of the effects of T1D on sperm physiology was extended to PREP, RSPH6A, and Dynein Axonemal Light Chain 1 (DNAL1), MAPs involved in sperm motility (Figure 8).

In line with several previous observations, T1D induced a significant decrease in PREP (*p* < 0.001), RSPH6A (*p* < 0.01), and DNAL1 (*p* < 0.05) protein levels, as compared to the control (Figure 8A).

Subsequent IF staining of SPZ supported these observations, showing that PREP (Figure 8B), RSPH6A (Figure 8C), and DNAL1 (Figure 8D) clearly localized in the tail, in co-localization with TUB. Interestingly, the DNAL1 signal also appeared in the dorsal and apical regions of the sperm head, at the acrosomal region. Fluorescence intensity analysis revealed a statistically significant trend consistent with the observed protein expression levels.

## 3. Discussion

Over the past decades, the global prevalence of T1D has steadily increased. Although traditionally classified as a childhood-onset disease, current epidemiological data indicate that adult diagnoses now surpass those in children [57]. T1D, often occurring alongside other metabolic disturbances, is a well-established contributor to OS. Chronic hyperglycemia in T1D triggers abnormal metabolic pathways that drive the excessive production of ROS, while simultaneously impairing both enzymatic and non-enzymatic antioxidant mechanisms. This results in a cellular redox imbalance that favors a pro-oxidant environment [58]. Such conditions can lead to structural and functional damage of various macromolecules and organelles. The cytoskeleton is one of the key cellular components susceptible to oxidative damage, with its dynamic organization particularly affected. This vulnerability has been documented across multiple cellular systems [59,60,61], and is especially evident in neurons [62], where MTs are essential for axonal elongation and vesicular transport.

This study provides new insight into how T1D-induced OS may impact MTs dynamics and the function of MAPs in the rat testis and SPZ, potentially contributing to male subfertility. Indeed, many cellular processes occurring during spermatogenesis rely on dynamic regulation of the cytoskeleton, including MTs nucleation, polymerization, depolymerization, and stabilization, processes influenced by MAP activity. MAPs are generally classified into two groups: (1) structural MAPs, which stabilize MTs, (2) motor proteins, which transport different cargoes, including GCs, along MT-based tracks [25]. MAPs are present in nearly all mammalian cells, and are especially abundant in SCs, where they regulate MTs reorganization during the epithelial cycle to support spermatogenesis. Despite their relevance, comprehensive studies investigating MAPs function in the testis remain limited; however, most available data derive from “toxicant models”, via in vitro or in vivo exposure to environmental toxicants [27]. Building on this background, we applied a similar integrative approach—combining molecular, cellular, and functional analyses to examine the expression and localization of several MAPs, such as MARK4, MAP1A, DYNLL1, PREP, and RSPH6A, in a rat model of T1D, all of which are involved in cytoskeletal regulation during spermatogenesis and sperm motility. To our knowledge, no omics datasets are currently available that directly examine the expression of MAPs in diabetic testicular tissue or sperm. Nevertheless, recent transcriptomic and multi-omics studies have described diabetes-induced molecular changes in male reproductive organs, particularly involving redox regulation and cell signaling [39,40,41,42]. These findings align with the broader mechanisms observed in our model and suggest a shared pathophysiological basis.

MARK4 is a Ser/Thr kinase regulating MTs dynamics via the phosphorylation of structural MAPs such as MAP1A [43,63]. MAP1A stabilizes MTs and promotes TUB polymerization, but phosphorylation by MARK4 can lead to its detachment, destabilizing MTs [63]. Their interplay supports proper MTs regulation during spermatogenesis [25,43,44,45]. In our study, both MARK4 and MAP1A protein levels and localization appeared altered in the testes of T1D animals. Interestingly, in controls, MARK4 localized at the basal BTB, whereas MAP1A was distributed in SCs cytoplasm, forming a track-like structures perpendicular to the basal membrane of the SE. In the gonads of T1D animals, both proteins assumed a more diffused cytoplasmic distribution in SCs, probably causing aberrant phosphorylation of MAP1A and reduced MTs stabilization, potentially contributing to MTs disorganization.

GCs are immotile and rely on SCs for translocation across the BTB via the apical ES, which moves along MTs tracks powered by motor proteins like dynein and kinesin [64,65]. Among these, dynein plays a key role in retrograde transport, moving cargoes toward the minus (−) ends of the MTs, located at the center of the cell or the basal compartment of the SE [23]. Specifically, dynein 1, of which DYNLL1 represents a light chain subunit, is involved in several physiological functions, including intracellular transport, cell polarization, mitosis, and SPT translocation [23]. Herein, we observed altered testicular expression and localization of DYNLL1 in elongating SPT in T1D animals, which may interfere with the proper transport and release of these cells into the lumen. Interestingly, a previous work has shown that dynein 1 knockdown by RNAi in the rat testis perturbs the BTB permeability and affects the distribution of junctional proteins such as ZO-1 and N-cadherin [66]. Based on our previous findings suggesting that T1D is associated with BTB impairment [36], here we hypothesize that the observed changes in DYNLL1 may be associated with this BTB dysfunction; however, a direct mechanistic link remains to be established.

Given the MTs alterations in T1D animals, we assessed SPZ functionality. We found increased 4-HNE, a lipid peroxidation marker that forms damaging protein adducts [67]. SPZ are particularly ROS-sensitive due to low antioxidant defenses and PUFA-rich membranes [68]. The observed OS may contribute to the impairment of several sperm parameters, including reduced number, altered morphology, decreased vitality and motility, and increased apoptosis, as supported by the elevated pro-apoptotic markers and a higher percentage of TUNEL-positive SPZ in T1D animals. This work also sheds light on chromatin remodeling defects in SPZ from T1D rats, with a significant increase in immature sperm exhibiting histone retention and abnormal protamine-to-histone exchange. Since protamines are critical for sperm DNA condensation and protection from oxidative damage, their reduced levels may predispose to a loss of genome integrity. These findings are corroborated by an increased prevalence of DNA fragmentation, as evidenced by AO staining, suggesting that molecular alterations observed in T1D may be associated with reduced gamete quality.

In line with the biochemical data, 4-HNE immunolocalization revealed a strong signal in the flagellar and acrosomal regions of SPZ. Previous data from our group demonstrated significant alterations in antioxidant enzymes and redox-sensitive pathways (e.g., KEAP1-Nrf2, NF-κB) in the same T1D model [36], supporting the broader involvement of OS responses in testicular dysfunction. These pathways may also represent therapeutic targets worthy of further investigation. Sperm motility depends on ATP, Ca^2+^ signaling, and an intact MTs-based flagellar structure. The initiation and maintenance of SPZ motility requires a large quantity of ATP [69] that is produced either via the glycolytic pathway and mitochondrial oxidative phosphorylation [70]. In our study, the reduced mitochondrial mass and ATP synthesis observed in the gametes of T1D rats suggest possible mitochondrial dysfunction, potentially resulting from oxidative damage. The positive correlation between mitochondrial activity and sperm quality has also been demonstrated in humans, where high mitochondrial function correlates with increased success in in vitro fertilization outcomes [71]. The relationship between mitochondrial function and sperm motility is well established in the literature [72,73,74]; however, while our findings align with this body of evidence, we recognize that additional functional studies are necessary to confirm a direct mechanistic role for mitochondrial impairment in T1D-associated sperm defects.

Ca^2+^ ions play a central role in several events preceding fertilization, particularly sperm motility, via the activation of downstream signaling pathways, including those involving cAMP [74]. The functional relevance of Ca^2+^ has also been demonstrated by studies showing that the disruption of Ca^2+^ signaling is associated with male subfertility [75]. SPZ use two main sources of Ca^2+^: intracellular stores, located in the acrosome and mitochondria, and extracellular influx. In both cases, Ca^2+^ enters the sperm cytoplasm through specific channels, such as VDAC3 [76] and CATSPER [77], respectively. In line with previous findings [78], our data indicate altered Ca^2+^ homeostasis in the T1D group, which may contribute to impaired motility, reduced viability, and compromised sperm function.

The flagellum of SPZ houses the structural machinery required for movement, known as the axoneme, which consists of nine MTs doublets arranged around a central pair [79]. Several proteins associated with flagellar MTs, and sperm motility include PREP, RSPH6A, and axonemal dynein. PREP is a serine protease with two roles: degradation of small peptides and modulation of MTs dynamics. RSPH6A belongs to the radial spoke protein family and connects the central and peripheral MTs pairs. The dynein arm motor complex, which includes DNAL1 as a subunit, mediates MTs sliding in an ATP-dependent manner, a critical process for motility. In our study, the altered expression and localization of these proteins were observed in T1D animals, not only in SPZ but also—specifically for PREP and RSPH6A—in testicular germ and somatic cells. These results align with previous work showing PREP and RSPH6A downregulation in conditions like OS, myotonic dystrophy, and asthenozoospermia.

In this complex scenario of T1D-associated alterations in MT dynamics and sperm motility, we also examined TUB acetylation, specifically at the amino group of lysine-40 in its N-terminal domain [80]. This post-translational modification has been implicated in regulating MTs structure, stability, and sperm motility [81] and lower levels of K-TUB have been reported in the sperm tails of asthenozoospermic men [81,82]. Many efforts have been made to identify the enzymes regulating the grade of TUB acetylation, and ATAT1 [55] and HDAC6 [56] have been identified as the main acetyltransferase and deacetylase, respectively, in mammalian sperm. T1D sperm showed reduced K-TUB and ATAT1, and increased HDAC6, indicating a shift in the acetylation/deacetylation balance. These alterations may destabilize axonemal structure and impair motility. K-TUB and MAPs co-localization in the tail suggests they cooperate in maintaining flagellar function.

Altogether, our findings provide a comprehensive view of the molecular and structural cytoskeletal defects induced by T1D, contributing to impaired spermatogenesis and sperm function.

This study presents some limitations that should be acknowledged. First, the experimental model used here—streptozotocin-induced type 1 diabetes in rats—recapitulates several key features of the disease but may not fully reflect the complexity and heterogeneity of human diabetic pathology (e.g., genetic variability, disease duration, comorbidities, and differences in metabolic control). Second, while we demonstrated strong molecular and cellular associations between T1D-induced OS, cytoskeletal disruption, and impaired sperm quality, we did not include direct functional assessments of fertility, such as mating outcomes or fertilization success. Third, although our findings suggest a potential mechanistic link between altered expression of MAPs and testicular dysfunction, further studies using knockdown or overexpression strategies would be necessary to establish a causal relationship. Finally, due to the cross-sectional nature of the study design, we could not address the progression or potential reversibility of these changes over time (e.g., whether they may improve following glycemic control or therapeutic intervention). Future investigations should consider these aspects to deepen our understanding of diabetes-related male infertility.

## 4. Materials and Methods

### 4.1. Animals, Treatments, and Sample Collection

Ten adult male Wistar rats (*Rattus norvegicus*), aged two months and weighing 220 ± 18.97 g, were individually housed in stainless steel cages under controlled environmental conditions (2 h light/dark cycle, 24 ± 2 °C temperature, and 55 ± 20% relative humidity). To ensure adequate adaptation to the new conditions, rats were subjected to an acclimatization period of one week before the start of the experiments. Throughout the study, the animals had free access to sterile food and water. The rats were randomly divided into two groups: the control (C; *n* = 5), which received 5% citrate buffer solution (#211018; AppliChem GmbH, Darmstadt, Germany), and the treated group (T1D; *n* = 5) which received a single intraperitoneal (i.p.) injection (65 mg/kg body weight) of streptozotocin (#18883–66-4; Chem Cruz Biochemicals; Huissen, The Netherlands) dissolved in the same buffer [36,83]. Blood glucose levels were regularly monitored throughout the experimental period, as described in our previous study [36] and confirmed persistent hyperglycemia in T1D animals. After three months, the animals were anesthetized with an i.p. injection of chloral hydrate (#15307; Sigma-Aldrich; Milan, Italy) followed by the administration of a lethal dose of urethane (2 g/kg; #94300; Sigma-Aldrich; Milan, Italy) to induce cardiac arrest. Immediately following euthanasia, testes were excised, rinsed in pre-warmed phosphate-buffered saline (PBS; P3813; Sigma-Aldrich; Milan, Italy), and the left testes were fixed in Bouin’s solution (#HT10132; Sigma-Aldrich; Milan, Italy) for histological analysis, while the right ones were snap-frozen in liquid nitrogen and stored at −80 °C and biochemical assays. SPZ were collected by mincing the epididymides in PBS, followed by filtration and microscopic inspection to rule out somatic cell contamination. Subsequently, aliquots of the suspension were air-dried on microscope slides and stored at −20 °C for staining, while the remaining samples were centrifuged (1000× *g*, 15 min, 4 °C) and preserved at −80 °C for downstream molecular analyses.

All experimental procedures were approved by the Animal Ethics Committee of the University of Campania “L. Vanvitelli” of Naples and by the Italian Ministry for Health (protocol number 30/2021). Animal care complied with the Italian (D.L. 116/92) and European Commission (O.J. of E.C. L358/1 18/12/86) regulations on the protection of laboratory animals. All efforts were made to reduce both the animal number and suffering during the study.

### 4.2. Sperm Parameter Evaluation

#### 4.2.1. Epididymal Sperm Count, Motility, Viability, and Morphology

All the main sperm parameters were evaluated in accordance with previously established protocols [84,85]. For morphological analysis, the SPZ suspension was diluted in distilled water to a final volume of 20 mL. One to two milliliters of 1% eosin were added and the mixture was incubated at room temperature for 1 h. A drop of the stained suspension was then smeared onto a glass slide and examined under a light microscope (Axiostar Plus Zeiss; Carl Zeiss Microscopy GmbH, Jena, Germany) at ×40 magnification. A total of 300 SPZ per slide were examined, and the percentage of abnormal forms was calculated.

Sperm motility was assessed by diluting the SPZ suspension 1:10 in PBS. A 20 µL aliquot was placed in a Malassez counting chamber, and motile versus non-motile SPZ were counted under the same optical microscope at × 40 magnification. Motility was expressed as the percentage of motile spermatozoa out of the total counted (300 cells per sample).

#### 4.2.2. AB Staining

AB staining was used to assess the chromatin condensation defects in SPZ. Air-dried sperm smears were fixed in 4% PFA for 10 min at 4° C, rinsed in distilled water for 2 min, and stained with 5% aqueous AB in 4% acetic acid (pH = 3.5) for 5 min. Slides were then briefly rinsed in distillated water, counterstained with 0.5% eosin, and air dried. Approximately 300 SPZ per animal were evaluated under a light microscope, and results were expressed as the percentage of AB-positive cells (blue-stained nuclei).

#### 4.2.3. AO Staining

AO staining was performed to evaluate DNA integrity in SPZ, following the protocol described by Tejada et al. [86]. Slides were examined under a fluorescent microscope equipped with a UV lamp (Leica DM5000 B + CTR 5000; Leica Microsystems, Wetzlar, Germany) and images were acquired using IM 1000 software (version 4.7.0) and a Leica DFC320 R2 digital camera. SPZ with intact DNA showed green fluorescence, whereas those with fragmented DNA showed yellow to red staining depending on the degree of damage. For each animal, approximately 300 SPZ were counted, and results were expressed as the percentage of AO-positive cells (yellow/red nuclei).

#### 4.2.4. ATP and Ca^2+^ Assays in SPZ

Intracellular ATP levels in SPZ were measured using a commercial kit (#ab83355; Abcam, Cambridge, UK). Fluorescence was recorded at excitation/emission wavelengths of 535/587 nm, and ATP concentrations were expressed as nmol/μL.

Intracellular Ca^2+^ levels in SPZ were assessed using a commercial kit (#701220; Cayman Chemical Company, Ann Arbor, MI, USA). Absorbance was measured using a spectrophotometer at a wavelength range of 560–590 nm.

### 4.3. Protein Extraction and WB Analysis

Total proteins were extracted from testes and SPZ by homogenizing the samples in ice-cold RIPA lysis buffer (#TCL131; Hi Media Laboratories GmbH; Einhausen, Germany) supplemented with 10 µL/mL of protease inhibitor cocktail (#39102; SERVA Electrophoresis GmbH; Heidelberg, Germany). Homogenates were centrifuged at 14,000× *g* for 20 min at 4 °C, and the resulting supernatants were collected and stored at −80 °C until use. Protein concentrations were determined using the colorimetric Lowry assay. For WB analysis, 40 µg of total protein per sample were separated by SDS-PAGE (9–15% polyacrylamide; #A4983; AppliChem GmbH, Darmstadt, Germany) and processed as previously described [87]. Detailed information on the primary and secondary antibodies used is provided in Table S1. The protein bands were detected by chemiluminescence and quantified using ImageJ software (version 1.53 t; National Institutes of Health, Bethesda, MD, USA). Densitometric values were normalized to TUB. All the WB analyses were performed in triplicate.

### 4.4. RT-PCR Analysis

Total RNA was extracted from testis samples using RNA-Xpress Reagent (#MB601; HiMedia Laboratories GmbH; Einhausen, Germany) and processed according to previously published protocols [88]. The details of the primers used are listed in Table S2. The expression levels of the *Prep* and *Rsph6a* mRNAs were calculated based on the Prep/Tub and Rsph6a/Tub ratio, and results were expressed as optical density (OD) units. All the RT-PCR experiments were performed in triplicate.

### 4.5. IF Analysis on Testis and SPZ

Testicular tissue sections (5 µm thick) were dewaxed in xylene, rehydrated through a graded ethanol series, and processed as previously described [89]. SPZ were fixed with 4% PFA and treated as described in Minucci and Venditti [90]. Slides were incubated with specific primary and secondary antibodies (see Table S1) then mounted with Vectashield + DAPI (#H-1200–10; Vector Laboratories, Peterborough, UK) and coverslipped. Observation and image acquisition were carried out using the same fluorescence microscope described in Section 4.2.3. Densitometric analysis of fluorescence signal intensity and quantification of positive cells was performed using the Fiji plugin (version 3.9.0/1.53t) of ImageJ software. For each group, 30 tubules/animal (150 tubules total) per group and approximately 300 SPZ per animal were evaluated. All the IF experiments were performed in triplicate.

### 4.6. TUNEL Assay

To detect apoptotic SPZ, the TUNEL assay was performed using the DeadEnd™ Fluorometric TUNEL System (#G3250; Promega Corp., Madison, WI, USA), following the manufacturer’s instructions. The nuclei were counterstained with Vectashield + DAPI. Approximately 300 SPZ per slide were analyzed, and results were expressed as the percentage of green/light blue TUNEL-positive cells.

### 4.7. Statistical Analysis

Statistical analyses were performed using GraphPad Prism version 8.0 (GraphPad Software, San Diego, CA, USA). Comparisons between the control and T1D groups were made using the unpaired Student’s *t*-test. Data are expressed as mean ± standard error of the mean (SEM), and differences were considered statistically significant at *p* < 0.05.

## 5. Conclusions

In summary, this study highlights the potential impact of T1D-associated OS on MTs dynamics and associated proteins in the rat testis and SPZ. The observed alterations in MAPs expression, mitochondrial function, Ca^2+^ homeostasis, chromatin integrity, and sperm motility-associated factors offer new insights into the molecular mechanisms potentially contributing to male subfertility in the context of T1D. Future studies incorporating bioinformatic analyses may help further clarify the molecular interactions and signaling pathways underlying MAPs-related alterations. Although no directly comparable omics datasets are currently available for testicular MAPs in T1D models, recent transcriptomic and multi-omics studies in diabetic contexts provide supportive evidence of OS-related dysregulation in male reproductive function. Notably, transcriptomic data from STZ-induced diabetic rat testes revealed altered miRNA–mRNA regulatory networks affecting Leydig cell function, including apoptosis and testosterone biosynthesis pathways [39,40,41,42]. While these data do not directly overlap with the cytoskeletal proteins analyzed here, they provide independent support for diabetes-induced molecular disruption in the male reproductive system. Collectively, these insights reinforce the relevance of our findings and highlight the importance of future integrative multi-omics approaches. Overall, our study underscores the critical role of cytoskeletal integrity in male reproductive health and supports the development of targeted therapeutic strategies to counteract diabetic subfertility.

## Figures and Tables

**Table 1:** Effect of T1D on rat sperm parameters.

Groups	C	T1D
SPZ concentration (10^6^/mL)	77.92 ± 5.87	53.45 ± 4.15 *
Viability (%)	82.22 ± 7.99	54.64 ± 8.08 **
Abnormal morphology (%)	16.37 ± 7.3	39.72 ± 6.61 ***
Motility (%)	80.08 ± 5.96	49.51 ± 4.51 ***

Evaluation of sperm parameters of T1D rats. Values are expressed as mean ± SEM from five animals in each group. *: *p* < 0.05; **: *p* < 0.01; *** *p* < 0.001.

## Data Availability

The original contributions presented in this study are included in the article. Further inquiries can be directed to the corresponding author.

## Supplementary Materials

The following supporting information can be downloaded at: https://www.mdpi.com/article/10.3390/ijms26104579/s1.
