# Supplementary material for: Impact of Type 1 Diabetes on Testicular Microtubule Dynamics, Sperm Physiology, and Male Reproductive Health in Rat
Source: Int J Mol Sci. 2025 May 10;26(10):4579. doi: 10.3390/ijms26104579 (PMC12111744; doi:10.3390/ijms26104579)
Supplement: Supplementary file 1 [file ijms-26-04579-s001.zip › Table S1.pdf]

Table S1. List of all the used antibodies.

Antibody	Molecular weight (kDa)	WB Dilution	IF Dilution	Source
MARK4	100	1:3000	1:100	Thermo Fisher Scientific, Waltham, MA, USA<br>#PA5-104542
MAP1A	250	1:1000	1:100	Thermo Fisher Scientific, Waltham, MA, USA<br>#PA5-106941
DYNLL1	10	1:1500	1:50	ABclonal Science, Inc. Woburn, MA, USA<br>#A4353
PREP	80	1:3000	1:100	Abcam, Cambridge, UK<br>#ab58988
RSPH6A	80	1:3000	1:100	Sigma-Aldrich, Milan, Italy<br># HPA045382
Histone H3	17	1:1000	-	Merck Millipore, Milan, Italy<br>#06–755
PRM2	13	1:500	-	Proteintech, Manchester, UK<br>#14500-1-AP
4-HNE	66	1:1000	1:100	Thermo Fisher Scientific, Waltham, MA, USA #BS-6313R
TOMM20	-	-	1:100	Sigma-Aldrich, Milan, Italy #WH0009804M1
P53	43	1:500	-	Elabscience Biotechnology, Wuhan, China<br>#E-AB-32469
BAX	21	1:750	-	Elabscience Biotechnology, Wuhan, China<br>#E-AB-13814
Bcl-2	22-26	1:750	-	Elabscience Biotechnology, Wuhan, China<br>#E-AB-60012
CATSPER	80	1:1000	1:100	Thermo Fisher Scientific, Waltham, MA, USA # BS-23327R

VDAC3	31	1:2000	1:100	ABclonal Science, Inc. Woburn, MA, USA #A26244
K-TUB	52	1:1000	1:100	Abcam, Cambridge, UK #ab179484
ATAT1	48	1:1000	1:50	Thermo Fisher Scientific, Waltham, MA, USA #PA5-114922
HDAC6	131	1:750	1:50	GeneTex, Irvine, CA, USA #GTX100722
DNAL1	22	1:1000	1:50	ABclonal Science, Inc. Woburn, MA, USA #A8267
α-Tubulin	52	1:5000	1:100	Elabscience Biotechnology, Wuhan, China #E-AB-20036
Goat anti-rabbit IgG HRP	-	1:5000	-	Vector Laboratories, Burlingame, CA, USA #PI-1000
Goat anti-mouse IgG HRP	-	1:5000	-	BioActs, Namdong-gu, Incheon, Korea #RSA1122
Goat anti-rabbit Alexa Fluor 488	-	-	1:500	Thermo Fisher Scientific, Waltham, Ma, USA #A32731
Goat anti-mouse CF™ 568	-	-	1:250	Sigma-Aldrich, Milan, Italy #SAB4600082
PNA lectin Alexa Fluor 568	-	-	1:50	Thermo Fisher Scientific, Waltham, Ma, USA #L32458
