# Supplementary material for: Impact of Type 1 Diabetes on Testicular Microtubule Dynamics, Sperm Physiology, and Male Reproductive Health in Rat
Source: Int J Mol Sci. 2025 May 10;26(10):4579. doi: 10.3390/ijms26104579 (PMC12111744; doi:10.3390/ijms26104579)
Supplement: Supplementary file 1 [file ijms-26-04579-s001.zip › Table S2.pdf]

Table S2. List of all the used primers.

Gene	GenBank Accession Nr.	Annealing Temperature	Amplicon size (bp)	Primers Sequence
Prep	NM_031324.2	55° C	392	For: 5′- CCCTTATGCTTGGCTTGAAG -3′<br>Rev: 5′- TCATGAACTTGATGGTCACC -3′
Rsph6a	NM_001415788.1	56° C	366	For: 5′- AGGAGAACCCTGACTTTGAG -3′<br>Rev: 5′- TCTCAAACTTCTTGCCAGTG -3′
α-Tubulin	AH002269.2	58° C	378	For: 5′- AAGACCAAGCGTACCATCCA -3′<br>Rev: 5′- TGAAAGCAGCACCTTGTGAC-3′
